# Efficacy of Secukinumab for Plaque Psoriasis in a Patient on Hemodialysis

**DOI:** 10.1007/s13730-019-00426-z

**Published:** 2019-10-25

**Authors:** Daiuske Ikuma, Masahiko Oguro, Junichi Hoshino, Hiroki Mizuno, Akinari Sekine, Masahiro Kawada, Rikako Hiramatsu, Keiichi Sumida, Eiko Hasegawa, Noriko Hayami, Masayuki Yamanouchi, Tatsuya Suwabe, Naoki Sawa, Kenmei Takaichi, Yoshifumi Ubara

**Affiliations:** Nephrology Center, Toranomon Hospital, 2-2-2Minato-ku, ToranomonTokyo, 105-0001 Japan

**Keywords:** Plaque psoriasis, Secukinumab, Hemodialysis and end stage renal disease (ESRD)

## Abstract

Secukinumab is effective to treat plaque psoriasis. However, the safety and efficiency of secukinumab have not been clarified in patients on hemodialysis. We report a 60-year-old Japanese woman. Plaque psoriasis was diagnosed at the age of 25 years and hemodialysis was started at the age of 39 years. Her skin lesions persisted despite use of topical agents such as maxacalcitol and betamethasone. Accordingly, administration of secukinumab was started at a dose of 150 mg. The psoriasis area and severity index (PASI) score decreased from 49.8 to 14.8 after 2 weeks and to 0 after 6 weeks, with remission being maintained after 28 months. No adverse reactions were seen. This case indicates that secukinumab may be effective for severe psoriasis in patients on hemodialysis for end-stage renal disease.

## Introduction

Plaque psoriasis is an autoimmune disease characterized by skin lesions that can reduce the quality of life, and it is considered to be associated with inflammatory cytokines, such as tumor necrosis factor (TNF)-α, interleukin (IL)-17, and IL-23 [1]. Recently, biological agents targeting these cytokines have been used to treat patients with plaque psoriasis. Secukinumab is a human monoclonal IgG1 antibody that blocks IL-17A activity and was reported to be effective for plaque psoriasis [2]. Large molecule such as immunoglobulin are only filtered to a very small extent by the kidneys [3]. Other biological agents are used without dose reduction in patients with renal dysfunction, so it is expected that secukinumab can be administered to these patients at the standard dose. However, the safety and efficiency of secukinumab have not been clarified in patients on hemodialysis.

Here we describe the efficacy and safety of secukinumab for severe plaque psoriasis in a patient on hemodialysis for end-stage renal disease.

## Case report

**Fig. 1:**
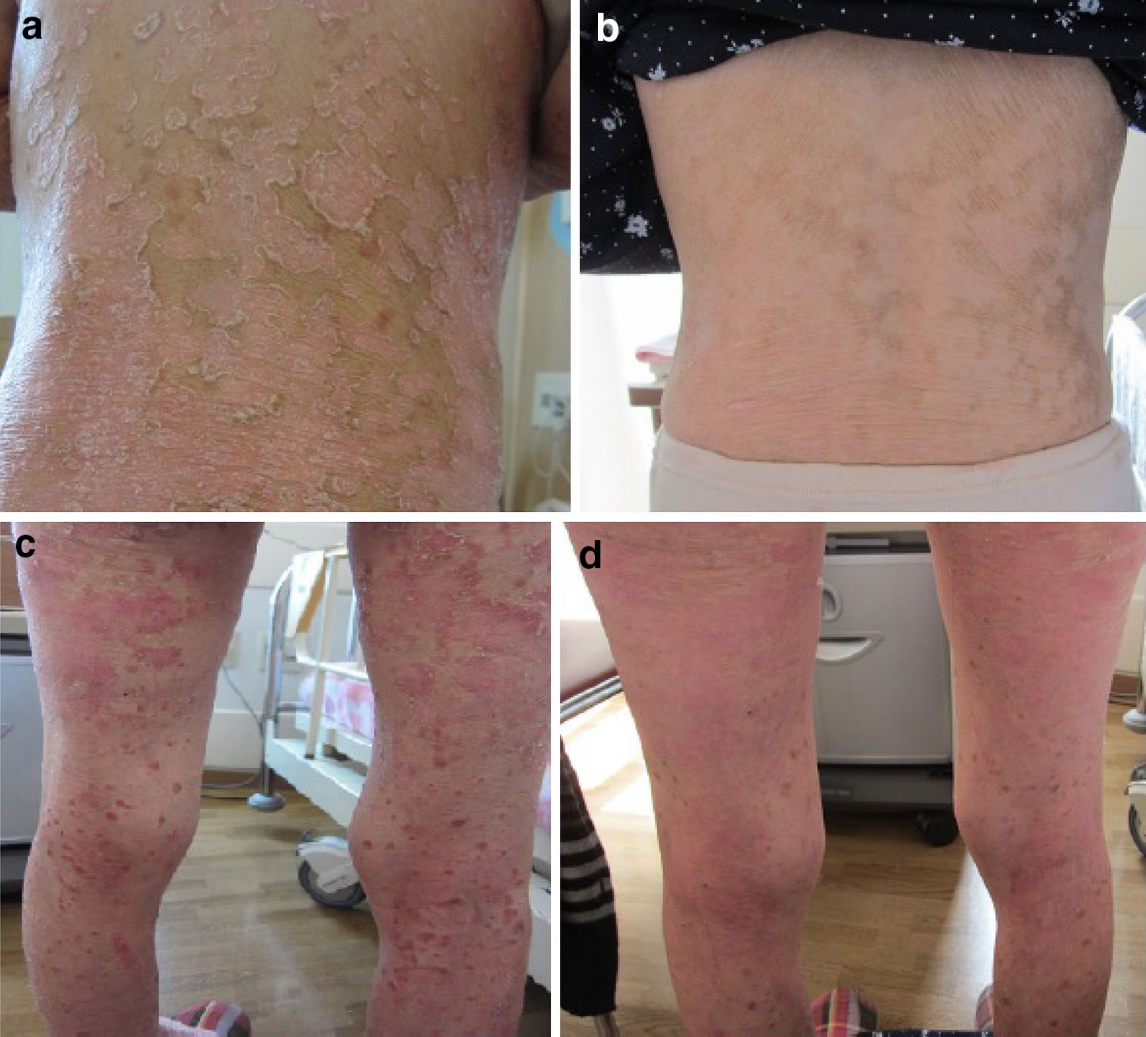
Clinical appearance. **a** Skin involvement of back before starting the treatment with secukinumab. **b** Skin involvement of back after 2 weeks of secukinumab. **c** Skin involvement of legs before starting the treatment with secukinumab. **d** Skin involvement of legs after 2 weeks of secukinumab

A 60-year-old Japanese woman was admitted to our hospital for evaluation of plaque psoriasis, which was initially diagnosed at the age of 25 years. Although she had used topical agents such as maxacalcitol and betamethasone, the skin lesion had persisted. Hemodialysis was started at the age of 39 years for chronic renal failure due to polycystic kidney disease. Four months before this admission, renal transcatheter arterial embolization was performed to treat enlargement of the kidneys, as reported previously [4]. Her skin lesions became worse three months later and the patient was admitted to our hospital. On admission, she was 147.0 cm tall and weighed 36 kg, with a blood pressure of 123/66 mmHg, heart rate of 70/min, and body temperature of 37.0℃. Raised, red and scaly patches, consistent with psoriatic plaques, were note on the skin of almost of all parts of the body (Fig. 1a, c). The psoriasis area and severity index (PASI) score was calculated to be 49.8 according to Fredriksson’s classification [5]. She had no pain or swelling of any joints. Laboratory tests showed that the leukocyte count was 4400/μL (76.5% neutrophils, 16.5% lymphocytes, and 1.8% eosinophils), hemoglobin was 11.0 g/dL, platelet count was 22.0 × 10^4^/μL, urea nitrogen was 56.0 mg/dL, serum creatinine was 7.79 mg/dL, C-reactive protein (CRP) was 0.1 mg/dL, and matrix metalloproteinase-3 (MMP-3) was 91.5 ng/mL (normal range 36.9–121). In addition, liver function and electrolyte levels were normal. Computed tomography revealed polycystic kidneys and the metal microcoils used previously for embolization.

**Fig. 2:**
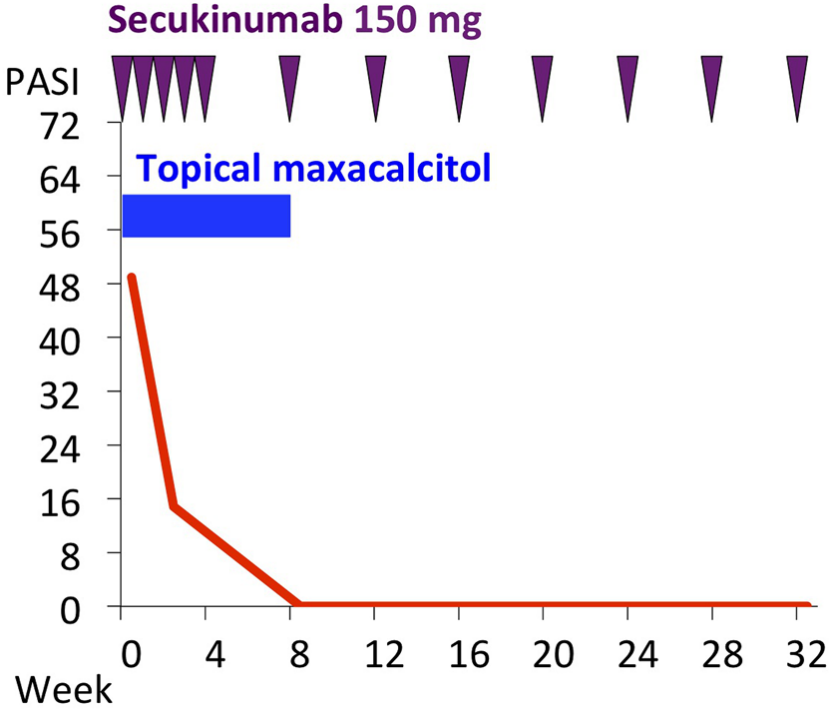
Clinical course. *PASI* psoriasis area and severity index

Secukinumab was started at a dose of 150 mg (half of the standard dose in consideration of her low body weight < 60 kg) by weekly injection. After four doses, treatment was changed to monthly injections. Her skin lesion was improved in after 2 weeks of secukinumab (Fig. 1b, d). PASI decreased to 14.8 after 2 weeks of treatment and to 0 after 6 weeks (Fig. 2), and it remains at zero after 28 months. She had used topical maxacalcitol at the start of secukinumab. The topical maxacalcitol was discontinued after 8 week because of improvement of skin lesion. No adverse events were observed, including infections.

## Discussion

Secukinumab was reported to achieve 82.8% of patients with plaque psoriasis greater than PASI-70 response at week 12 [6]. On the other hands Infliximab, adalimumab and ustekinumab achieve respectively 68.6%, 53.5% and 59.4% of patients with plaque psoriasis PASI-70 [7–9]. Secukinumab may act more quickly than infliximab, adalimumab and ustekinumab. Our patient’s skin lesion was severe and progressive. Thus we selected this biologic agent.

Secukinumab is a biological agent (monoclonal IgG) and is mainly metabolized in the reticuloendothelial system [10], so its blood level is not expected to be affected by hemodialysis. In fact, it was reported that the blood concentration of etanercept, a fusion protein combining an IgG1 antibody and the TNF receptor, was similar before and after hemodialysis [11]. The efficacy and safety of biological agents have been established in patients with psoriasis and end stage renal disease (Table 1). Saougou et al. presented a patient with severe psoriasis and dactylitis on chronic renal failure requiring regular hemodialysis had been improved both skin lesion and dactylitis by treatment if infliximab [12]. Cassano et al. reported the successful use of etanercept in a 69-year-old man with widespread psoriasis and end stage renal disease as a result of autosomal dominant polycystic kidney disease [13]. Kusakari et al. reported a 46-year-old Japanese man on hemodialysis who was treated with adalimumab for severe psoriasis and achieved a PASI-100 response at 2 months with no adverse effects after 1 year [14]. Moreover, Umezawa et al. administered ustekinumab to three psoriasis patients on hemodialysis and reported improvement at 1 year without adverse events [15]. Thus, a standard dosage of secukinumab would be expected to show efficacy in patients on hemodialysis.

**Table 1:** Summary event of biologics in patients with end stage renal disease in literature

References	Age	Sex	Disease	Duration (week)	Biologics	Outcome (skin lesion)	Outcome (arthritis)	Adverse event
Saugou et al.	52	M	Psoriatic arthritis	24	Infliximab	Effective	Effective	None
Cassano et al.	69	M	Psoriatic arthritis	24	Etanercept	Effective	Effective	None
Kusakari et al.	46	M	Psoriasis	48	Adalimumab	Effective	–	None
Umezwa et al.	68	M	Psoriasis	52	Ustekinumab	Effective	–	None
	64	M	Psoriasis	52	Ustekinumab	Effective	–	None
	57	M	Psoriasis	40	Ustekinumab	Effective	–	None

Our patient did not develop any adverse events during 28 months of treatment. Sumida et al. reported that renal insufficiency did not increase the risk of adverse events in patients receiving adalimumab for treatment of rheumatoid arthritis, and that adalimumab did not cause deterioration of renal function in patients with or without renal insufficiency [16].

In conclusion, we demonstrated that secukinumab was effective for plaque psoriasis without causing any serious adverse events in a patient on hemodialysis. This case suggests that secukinumab could be a useful therapeutic option for patients with severe plaque psoriasis and end-stage renal disease.
